# Bayesian modelling of the fossil record enlightens the evolutionary history of Hemiptera

**DOI:** 10.1098/rspb.2025.1133

**Published:** 2025-09-10

**Authors:** Mathieu Boderau, Yanzhe Fu, Hui Jiang, Shihan Guan, Ancheng Peng, Andre Nel, Corentin Jouault

**Affiliations:** ^1^ Institut de Systématique, Évolution, Biodiversité (UMR 7205), Muséum national d’Histoire naturelle, CNRS, Sorbonne Université, EPHE-PSL, Université des Antilles, Paris F-75005, France; ^2^ State Key Laboratory of Palaeobiology and Stratigraphy, Nanjing Institute of Geology and Palaeontology, Chinese Academy of Sciences, Nanjing, People’s Republic of China; ^3^ Department Paleontology, Bonn Institute of Organismic Biology (BIOB), University of Bonn, Bonn 53115, Germany; ^4^ Institute of Geology and Paleontology, Charles University, Prague 12843, Czech Republic; ^5^ Senckenberg Forschungsstation Grube Messel, Senckenberg Forschungsinstitut und Naturmuseum Frankfurt/M, Messel, Germany; ^6^ Laboratoire Traitement du Signal et de l’Image (UMR 1099), Université de Rennes, INSERM, Rennes F-35000, France; ^7^ Oxford University Museum of Natural History, University of Oxford, Parks Road, Oxford OX1 3PW, UK

**Keywords:** evolution, extinction, Insecta, macroevolution, palaeoenvironment, true bugs

## Abstract

Hemiptera, the fifth most diverse insect order, are characterized by their high diversity in deep time, with 145 known extinct families. However, the precise timing of the origin of Hemiptera lineages has remained uncertain. Traditional approaches, molecular clock analyses and fossil calibrations, have overlooked much of this extinct diversity by failing to incorporate key fossil data. Furthermore, no estimates have been proposed for the timing of the extinction of Hemiptera’s fossil lineages. In this study, we use the recently developed Bayesian Brownian Bridge model, which estimates the timing of lineage origin and extinction through fossil-based Bayesian modelling, to provide a temporal framework for the rise and fall of 310 major hemipteran lineages. Our results support an early Pennsylvanian origin of Hemiptera, and indicate that the major hemipteran lineages originated between the late Carboniferous and Late Permian (Pennsylvanian-Lopingian). Additionally, our analyses reveal a radiation of Hemiptera during the Permian (Guadalupian), followed by multiple extinctions of ancient hemipteran lineages from the Permo-Triassic boundary to the mid-Triassic. A second major radiation occurred during the Cretaceous, coinciding with numerous extinctions of relic and newly emerging Cretaceous lineages, highlighting a faunal turnover. Our study provides a holistic fossil-based picture of the evolutionary history of Hemiptera.

## 1. Introduction

**Figure 1.**
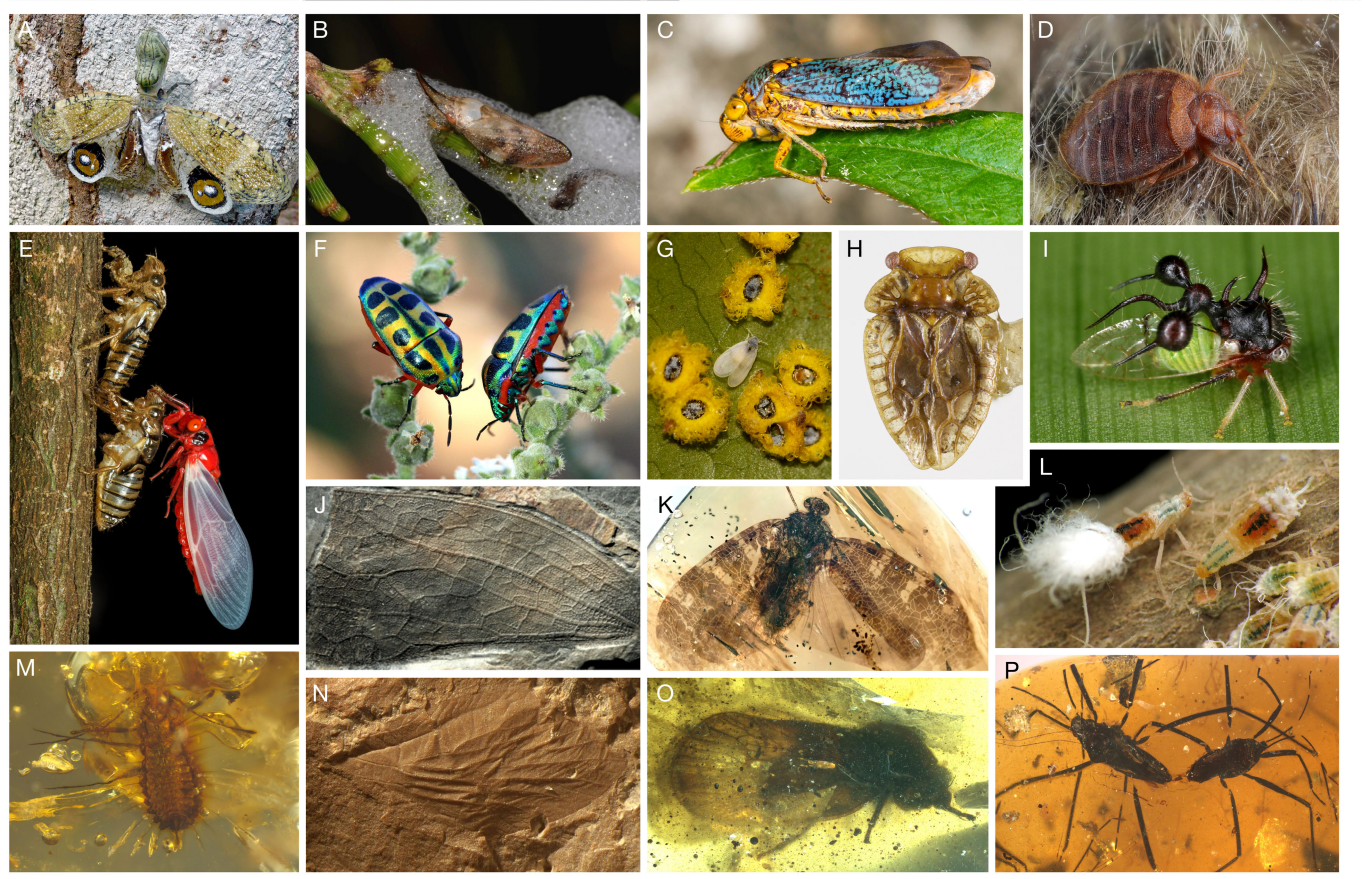
Past and present diversity of Hemiptera. (A) *Fulgora laternaria* Linnaeus, 1758 (Fulgoromorpha: Fulgoroidea: Fulgoridae). Photograph © Bernard Dupont. (B) *Philagra parva* Donovan, 1805 (Cicadomorpha: Cercopoidea: Aphrophoridae). Photograph © Nick Lambert. (C) *Oncometopia orbona* Fabricius, 1798 (Cicadomorpha: Membracoidea: Cicadellidae). Photograph © Ashley M Bradford. (D) *Cimex lectularius* Linnaeus, 1758 (Heteroptera: Cimicoidea: Cimicidae). Photograph © Gilles San Martin. (E) *Huechys sanguinea* De Geer, 1773 (Cicadomorpha: Cicadoidea: Cicadidae). Photograph © Ivan Neo. (F) *Calidea dregii* Germar, 1837 (Heteroptera: Pentatomoidea: Scutelleridae). Photograph © Cecile Roux. (G) *Aleurocanthus ceracroceus* Martin, 1999 (Sternorrhyncha: Aleyrodoidea: Aleyrodidae). Photograph © Nick Lambert. (H) *Xenophyes forsteri Drake and Salmon, 1948* (Coleorrhyncha: Peloridioidea: Peloridiidae). Photograph © Museum of New Zealand Te Papa Tongarewa. (I) *Cyphonia clavata* Fabricius, 1787 (Cicadomorpha: Membracoidea: Membracidae). Photograph © Elisabeth Glatzhofer. (J) *Permoridium fresenaci* Burckhardt, Nel, Raisch, Poschmann, 2022 (Coleorrhyncha: Permoridiidae). (K) *Multistria irregularis* Fabrikant, Huang and Fu, 2024 (Fulgoromorpha: Mimarachnidae). (L) *Grylloprociphilus imbricator* Fitch, 1851 (Sternorrhyncha: Aphidoidea: Aphididae). Photograph © Katja Schulz. (M) Scale insect from mid-Cretaceous Kachin amber (Coccoidea). (N) *Aviorrhyncha magnifica* Nel, Bourgoin, Engel, Szwedo, 2013 (Hemiptera: Aviorrhynchanidae). (O) *Eunotalia emeryi* Jiang *et al.* 2024 (Cicadomorpha: Cicadoidea, stem cicadoids). (P) *Burmogerris rarus* Fu, Cai, Chen and Huang, 2024 (Heteroptera: Gerroidea). Pictures without copyrights were taken by the authors.

Hemiptera, which include aphids, whiteflies, scale insects, jumping plant lices, moss bugs, leaf-, tree- and planthoppers, cicadas and true bugs, represent the fifth most species-rich insect order, with over 107 000 extant species [1, 2]. They exhibit remarkable behavioural, ecological and morphological diversity, with many lineages closely associated with plants for feeding (figure 1) [3], but also predatory and parasitic groups (e.g. assassin bugs and bed bugs) which are widely regarded as pests and vectors of human diseases [4]. While the extant or extinct diversity of Hemiptera is comparable to that of some other major insect orders, what sets them apart is their past diversity [5,6]. Hemiptera were hyper-diverse in deep time, with 145 recorded extinct families (figure 1) [5,7].

The evolutionary history of Hemiptera is complex, with diversity dynamics in deep time marked by alternating periods of diversification and extinction [6,8,9]. These fluctuations were often driven by major environmental changes, including mass extinctions such as the Permo-Triassic event and floral turnovers, like those observed during the Angiosperm Terrestrial Revolution [5,6,8,10]. While fossil-based studies have recently provided insights into the global patterns of hemipteran diversification over time [6], the precise timing of the origin of many Hemiptera lineages remains uncertain due to different estimates obtained in molecular clock analyses. A literal reading of the fossil record suggests that Hemiptera emerged over 325−315 million years ago (Ma), based on the oldest known fossil stem representatives, *Protoprosbole straeleni* Laurentiaux, 1952 and *Aviorrhyncha magnifica* Nel, Bourgoin, Engel, Szwedo, 2013 [11–13]. However, molecular clock studies have produced widely varying estimates for the origin of the clade and its constitutive lineages, highlighting the need for further investigation. Among the most optimistic scenarios, based on transcriptomic data, Hemiptera are estimated to have originated during the early Carboniferous (Mississippian), before 350 Ma [14] or during the Devonian (around 380 Ma) [15]. Other studies, using mitogenomic data, suggest a younger origin in the late Carboniferous (Pennsylvanian), before 310 Ma [16].

Beyond the discrepancies arising from different methodologies and data sources, the unique evolutionary history of Hemiptera, where extinct families nearly outnumber extant ones, suggests that a complete understanding of their evolution is impossible without considering their fossil diversity. Indeed, over 40% of Hemiptera families that once existed on Earth are now extinct [5]. This poses a challenge when estimating divergence times using traditional molecular clock and tree-based analyses. Most of these methods rely on fossils solely as node calibrations rather than integrating them as tips into the phylogeny [17–19]. This can result in long, bare branches within phylogenetic trees, a phenomenon known to introduce biases in analyses: [20–23], reflecting once-diverse lineages that are now represented by relatively few living species (e.g. Enicocephalomorpha) [15,24]. Additionally, the exclusion of key ‘fossil intermediates’ complicates the resolution of relationships and hinders accurate divergence time estimates [23,25,26]. Although methodological progress has improved the integration of fossil tips into phylogenies [27–29], they remain underutilized for reconstructing insect evolutionary history and have never been applied at a large scale for Hemiptera [30] (but see [31]). Therefore, it is expected that the evolutionary history of Hemiptera, encompassing many extinct lineages, and the timing of the origin and radiation of lineages cannot be accurately reconstructed using molecular data alone or without integrating extinct lineages into topologies.

As a result, alternative approaches that analyse fossil data within a Bayesian framework have emerged as powerful tools for reconstructing evolutionary scenarios and estimating the time of origin and extinction of groups [32–34]. Here, we apply the recently developed Bayesian Brownian bridge (BBB) model to estimate the timing of origin and extinction for 310 Hemiptera lineages with fossil representatives, leveraging the extensive fossil record of the order [32,33]. This model is well-suited for such analyses as it effectively accounts for biases inherent in the fossil record, such as increased sampling towards the present [32–34].

We present the first comprehensive study estimating the timing of origin for Hemiptera lineages, refining the time scale of their diversification and offering a complement to time-calibrated phylogenies, particularly for Hemiptera lineages that were more taxonomically diverse in deep time than they are today (e.g. Fulgoromorpha and Coleorrhyncha). Our study also provides the first estimates for the timing of extinction of major fossil lineages within Hemiptera, including emblematic extinct groups such as Prosbolomorpha. Ultimately, our results shed new light on the complex evolutionary history of the main hemipteran lineages (Sternorrhyncha, Cicadomorpha, Fulgoromorpha, Coleorrhyncha and Heteroptera), highlighting periods of faunal turnover that have shaped Hemiptera diversification.

## 2. Methods

### (a) Fossil record of Hemiptera

We used the dataset of Boderau *et al*. [6] as a starting point and created a new dataset of fossil species (electronic supplementary material, dataset 1). This study adopts the most comprehensive systematic framework for Hemiptera available to date, which integrates both extant and extinct lineages [5]. For Carboniferous hemipterans, we follow the classifications and arguments put forth by Boderau *et al*. [6], particularly regarding the placement of *Aviorrhyncha magnifica* [13] and *Protoprosbole straeleni* Laurentiaux, 1952. We kept one occurrence per species, except when a species is found in two deposits of different ages, in which case we kept one occurrence per species in each stage. When a species was found in two deposits of similar age, we retained only one occurrence. We then computed the ‘mean age of the species occurrence’ by considering both the lower and upper boundaries of the life span of every species occurrence. These data were then employed to compile the number of species into one-million-year time intervals (electronic supplementary material, dataset 2). This approach avoids artificially reducing or inflating the age of species when using the lower or upper boundary. This risk is particularly high for species from deposits with an age spanning two geological stages, such as the Dominican amber (Burdigalian–Langhian). The complete datasets used for each analysis are available in electronic supplementary material, datasets 1–9, for family level analyses (electronic supplementary material, data 2–3), for super-family level analyses (electronic supplementary material, datasets 4–5), for infra-order level analyses (electronic supplementary material, datasets 6–7), for sub-order level analyses (electronic supplementary material, datasets 8–9). The final dataset encompasses 3063 species of hemipterans spanning from the Pennsylvanian (Upper Carboniferous) to the Holocene.

### (b) The Bayesian Brownian bridge model

We analysed our dataset using the BBB model [32,33], which estimates the age of origin and extinction of groups based on a vector (representing the number of species through time per predefined time bin of 1 million years), the present day diversity of groups, and following the assumption that groups’ diversity evolves following a random walk bridging records of fossils species and anchored at both the starting point (first known species) and the ending point (last known fossil or extant diversity at present day). Details about the model can be found in Silvestro *et al*. [32] and Carlisle *et al*. [33].

### (c) Estimates of Hemiptera age and extinction

We applied the BBB model to the Hemiptera fossil record using a temporal constraint to the maximum boundary of the uniform prior on the root age (*-max_age* option). We ran our analyses under four different prior settings: 325 Ma (*-max_age* 325), 350 Ma (*-max_age* 350), 386 Ma (*-max_age* 386) and 407 Ma (*-max_age* 407). The first setting is based on the age of *P. straeleni*, the putative oldest hemipteran representative [5,6,13,35]. The second constrained age originates from the minimum age of radiation proposed for Hemiptera by Johnson *et al*. [15], and follows the results of Misof *et al*. [14] for the divergence between Thysanoptera and Hemiptera. The third maximum age corresponds to the radiation of Hemiptera estimated by Johnson *et al*. [15] and the fourth to the root prior age of the phylogenetic time-calibrated tree in Johnson *et al*. [15] after Misof *et al*. [14]. We recommend conducting multiple analyses using different prior settings for the *-max_age* parameter to assess the sensitivity of the results to this choice. As a preliminary guideline, we suggest using the age of the oldest known fossil (stem-group) representative of the clade as one option for -*max_age*. Additionally, we encourage exploring the influence of this parameter by incorporating alternative estimates of clade origin derived from molecular clock analyses and advise applying consistent maximum ages across all analyses.

We defined four datasets to estimate the time of origin and extinction of hemipteran at different systematic levels: (i) 228 families (electronic supplementary material, table S2); (ii) 55 superfamilies (electronic supplementary material, tables S1 and S3); (iii) 21 infraorders (electronic supplementary material, table S4); (iv) six suborders (electronic supplementary material, tables S5 and S6). Note that, for example, if a super-family contains only one family (e.g. Prosboloidea has only one family with Prosbolidae), the analyses are not performed at both levels, and the result of the smaller rank is used for the upper rank. For all our analyses, we used a preservation model with a time-increasing rate (*-q_var 1*), reflecting the increase in species number towards the present day of most extant clades [36,37].

Our dataset encompasses more than 310 groups for which we determined the age of origin and extinction (when applicable). When considering the four sets of analyses (i.e. with a different maximum boundary of the uniform prior on the root age), the number of analyses overpass 1,200. To alleviate the computational burden of running large‐scale BBB analyses, we pursued two complementary acceleration strategies. First, we parallelized the MCMC procedure by running multiple chains concurrently on separate CPU cores, exploiting the natural independence of Markov chains to expedite both sampling and convergence checks. Second, to handle particularly computationally intensive steps, we developed a separate method that offloads operations to GPU hardware, leveraging its high-throughput architecture for tasks such as large matrix manipulations. We evaluated the performance of the new scripts by analysing simulated datasets (electronic supplementary material, figure S1) corresponding to three simulated datasets: (i) 10 extant groups (*-sim 10*); (ii) five extant and five extinct groups (*-sim 5 -sim_extinct 5*) and (iii) 10 extinct groups (*-sim_extinct 10*). Since no explicit licence is stated for the distribution of the BBB model, we cannot publish this new script and simply encourage other users to adopt similar enhancements that significantly reduce computational burdens.

Each Hemiptera group was analysed independently using the BBB model that ran for 1 000 000 Markov chain Monte Carlo (MCMC) iterations and a sampling frequency of every 1000. We examined the results in Tracer 1.7.2 [38] to monitor *a posteriori* convergence of the chains. We considered that the parameters are convergent when ESSs are greater than 200, following Silvestro *et al*. [32]. In the discussion, we focussed on the results obtained using a -*max_age* set to 350 Ma, as this estimate is based on transcriptomic data [14,15], which are generally less affected by the accelerated evolutionary rates characteristic of mitogenomes, rates that can lead to artificially older divergence time estimates [39]. To assess the impact of different priors for the maximum allowed age, we also conducted statistical comparisons between results obtained under the four maximum age constraints defined above for the family-level dataset, which offers a sufficiently large sample size for such analyses (*n* > 50).

## 3. Results

**Figure 2.**
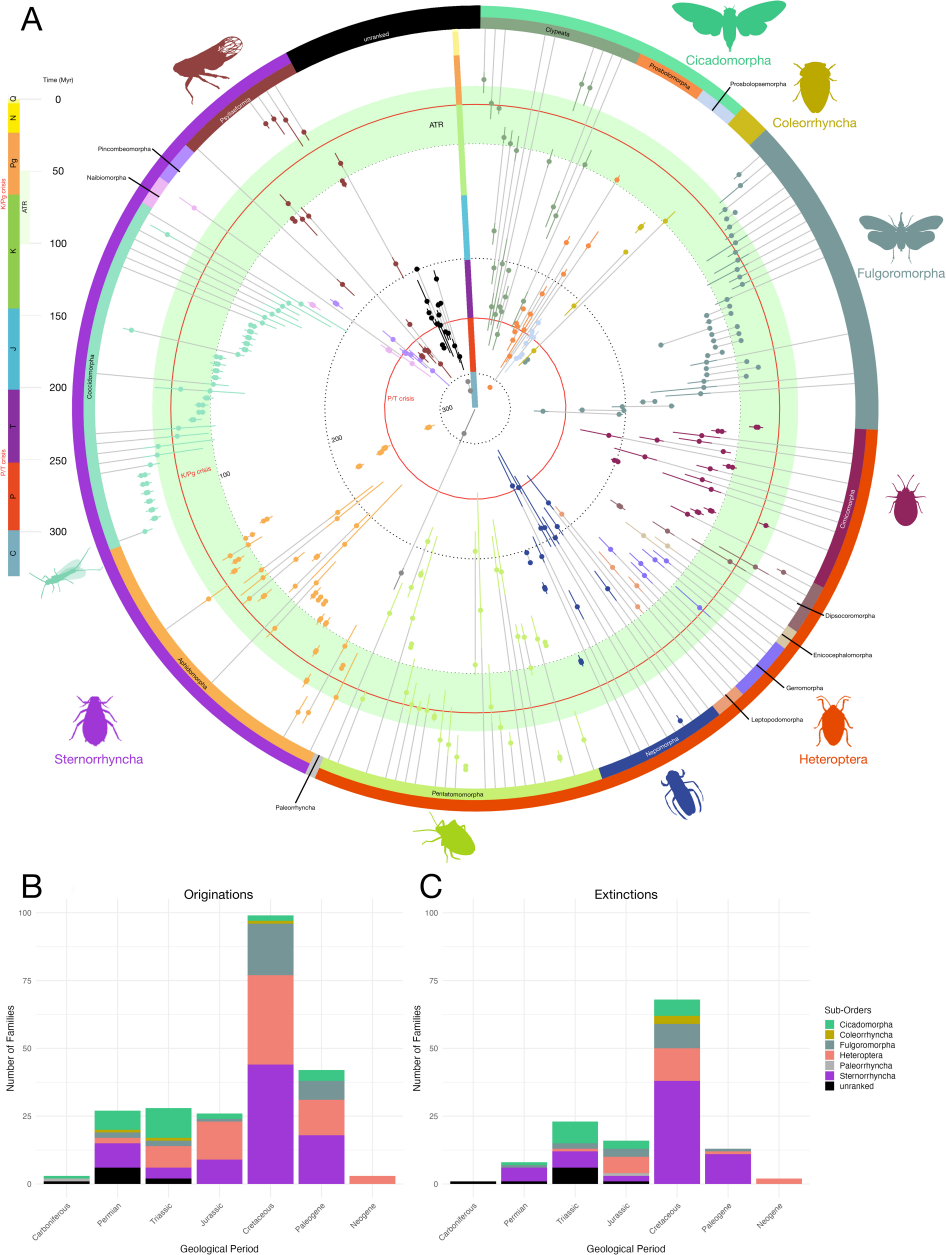
A time scale for the origin and extinction of Hemiptera families. (A). Clade age and extinction time estimates for Hemiptera families. Each line represents a family (arranged by sub-order then infra-order, if possible), with 95% credible intervals in colours at the root estimates and extinction estimates (where applicable). Grey lines correspond to the inferred lifespan of the lineage. Numbers correspond to time in millions of years (Myr). Radial coloured lines correspond to the geochronological scale with time in Myr. Red lines correspond to the two major Mesozoic mass extinction events: P/T: Permo-Triassic and K/Pg: Cretaceous/Palaeogene. Light green area corresponds to the Angiosperm Terrestrial Revolution. Time divided by geological periods with C: Carboniferous, P: Permian, T: Triassic, J: Jurassic, K: Cretaceous, Pg: Palaeogene, N: Neogene and Q: Quaternary. Hemiptera silhouettes from http://phylopic.org/licences at https://creativecommons.org/publicdomain/zero/1.0/ or drawn by Mathieu Boderau (Cicadomorpha, Coleorrhyncha and Nepomorpha silhouettes). (B) Number of originations estimated in each geological period per sub-orders. (C) Number of extinctions estimated in each geological period per sub-orders. Geological period bins are preferred to help reduce the impact of Lagerstätte effects, although the peak observed during the Cretaceous is likely influenced in part by the exceptional preservation of Burmese amber.

**Figure 3.**
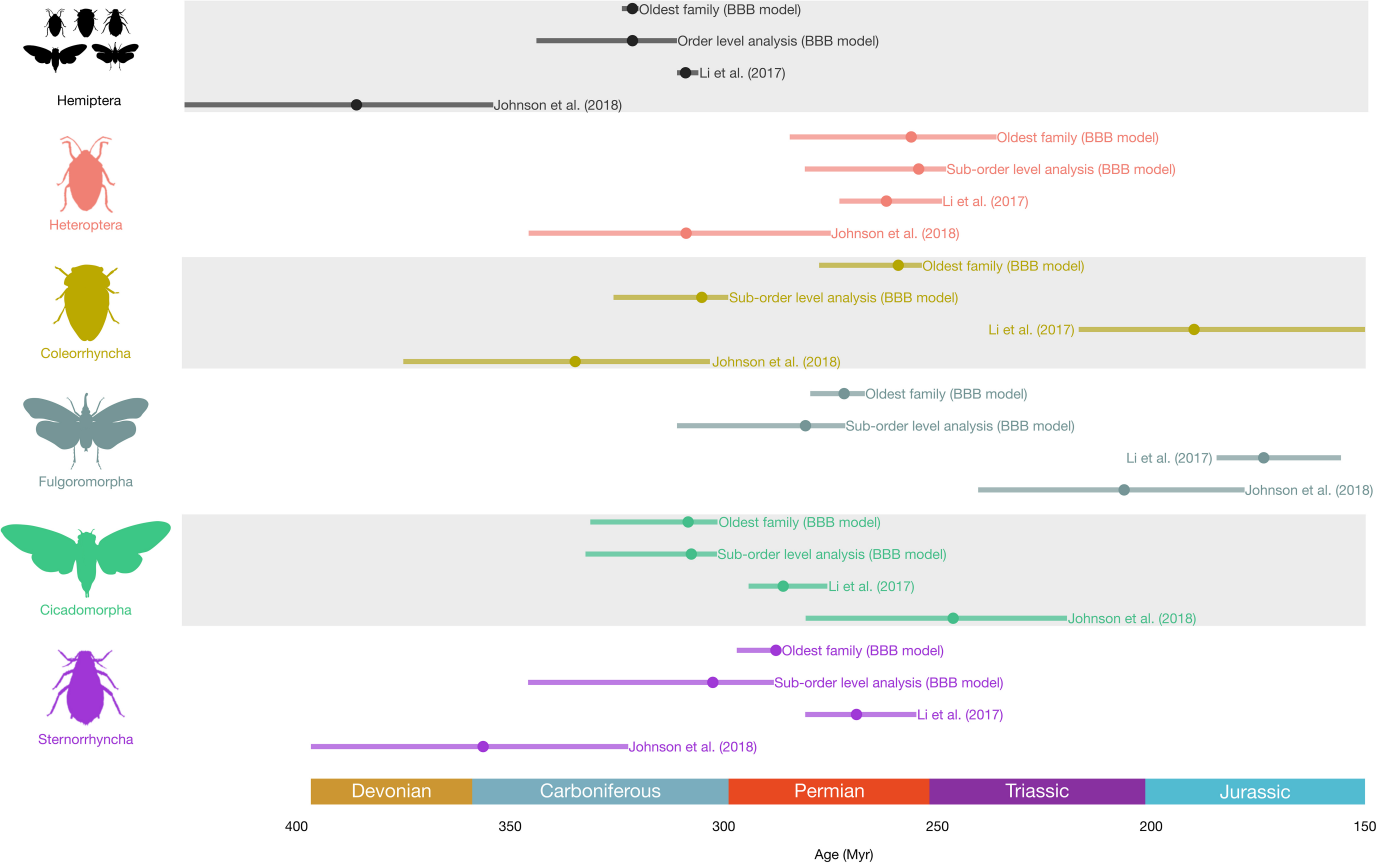
Ages of origin for Hemiptera and their major extant suborders estimated using the BBB model and compared to molecular-based divergence-time analyses [15,16]. Each line corresponds to an estimation for a clade with 95% credible intervals with the circles for the median values. Hemiptera silhouettes from http://phylopic.org/licences at https://creativecommons.org/publicdomain/zero/1.0 or drawn by Mathieu Boderau (Cicadomorpha and Coleorrhyncha).

Our analyses provide estimates for root and extinction ages for 310 Hemiptera lineages, even for lineages with fewer fossil representatives (figures 2 and 3).

Hemiptera began their diversification during the Carboniferous (figure 2A,B), with an origin during the Bashkirian (median 321.42 Ma, CI = 311−343.9 Ma), the oldest families being the extinct Protoprosbolidae (321.44 Ma, CI = 320−323.9 Ma), Aviorrhynchidae (314.79 Ma, CI = 314.01−317.45 Ma) and Archescytinidae (307.16 Ma, CI = 297.56−330.23 Ma), the latter belonging to the extinct suborder of Palaeorrhyncha. At a higher rank (figures 2A and 3), our analyses estimated that three hemipteran suborders originated during the Carboniferous: Cicadomorpha (307.65 Ma, CI = 301.64−332.42 Ma), Sternorrhyncha (302.62 Ma, CI = 288.38−345.85 Ma) and Coleorrhyncha (305.20 Ma, CI = 299.01−325.86 Ma).

The Permian (Guadalupian to Lopingian) is the first period of radiation for Hemiptera (figures 2A,B and 3; electronic supplementary material, table S2). Three main groups of Cicadomorpha: the monophyletic extant Clypeata, and the two extinct and possibly paraphyletic/polyphyletic Prosbolomorpha and Prosbolopsemorpha, originated during this period (electronic supplementary material, table S4). In particular, the Prosbolomorpha (306.95 Ma, CI = 301.28−325.98 Ma) are highly diversified during the late Palaeozoic to early Mesozoic, with notably the Prosbolidae (308.4 Ma, CI = 301.52−331.29 Ma). The Psyllaeformia, an extant major lineage of Sternorrhyncha, originated in the Permian (292.01 Ma, CI = 279.3−335.68 Ma), represented by the Protopsyllidioidea (298.76 Ma, CI = 283.07−335.99 Ma) with the Palaeozoic Lodevopsyllidae, Permopsyllidiidae and Protopsyllidiidae (electronic supplementary material, table S2).

During the Permian, the two last extant sub-orders are estimated to have originated: (i) the Fulgoromorpha (280.94 Ma, CI = 271.63−311 Ma), with the two major lineages Coleoscytidae and Fulgoroidea (electronic supplementary material, tables S1−S3), but only one family of the Fulgoroidea is recorded in the Palaeozoic; (ii) true bugs (Heteroptera) in the Late Permian (254.45 Ma, CI = 248.02−281.09 Ma), corresponding to the estimated apparition of true aquatic bugs (Nepomorpha; electronic supplementary material, table S4); and (iii) two euhemipteran extinct infra-orders are nested in the Permian: the Scytinopteromorpha (283.67 Ma, CI = 279.42−295.18 Ma) with five families originating from the Middle to Upper Permian (electronic supplementary material, table S2) and Ingruomorpha, represented only by the Ingruidae (274.86 Ma, CI = 274.01−276.14 Ma). This period also corresponds to the likely first records of the Coleorrhyncha with *Permoridium fresenaci* [40] as well as the estimated origin of the Progonocimidae (259.22 Ma, CI = 253.70−277.75 Ma), even if the attribution to the Coleorrhyncha is questionable [40] (but see also [41]).

Our analyses reveal multiple extinctions of Permian families close to the Permo-Triassic boundary (figure 2A,B). It slightly affected the Cicadomorpha at the family level with only the extinctions of Ignotalidae (251.23 Ma, CI = 247.362−251.97 Ma), Pereboriidae (250.97 Ma, CI = 243.72−252.89 Ma). In Sternorrhyncha, almost all Permian families went extinct, except the Naibiidae and Protopsyllidiidae (electronic supplementary material, table S2).

During the Triassic period, ‘relictual’ sternorrhynchan families went extinct (figure 2A), and the Triassophidoidea appeared (248.65 Ma, CI = 242−291.76 Ma) and went extinct before the end of the period (240.45 Ma, CI = 228.89−284 Ma). The true bugs started their radiation during the Triassic, with Pentatomomorpha, Cimicomorpha and Nepomorpha families (figure 2A). All Scytinopteromorpha went extinct by the start of the Jurassic, with major extinctions in the Early Triassic and only the Scytinopteridae and Ipsviciidae went extinct during the Early Jurassic (figure 2). Within the Cicadomorpha, extinctions are estimated during the Triassic for eight families (electronic supplementary material, table S2), with the Prosbolopsemorpha estimated to have gone extinct during the Middle Triassic (234.27 Ma, CI = 229.68−235.99 Ma).

During the Jurassic, Sternorrhyncha and Heteroptera radiated (figure 2; electronic supplementary material, tables S1−S3), while only three hopper families originated during this period (figure 2; electronic supplementary material, table S2). The heteropteran Gerromorpha are estimated to have originated during the Jurassic with the Mesoveliidae (160.9 Ma, CI = 154.05−175.65 Ma). Additionally, two other infra-orders of true bugs are estimated to have originated in the Jurassic: Dipsocoromorpha and Leptopodomorpha (electronic supplementary material, table S4). In Sternorrhyncha, the Jurassic corresponds to the extinction of the infra-order Pincombeomorpha (170.47 Ma, CI = 159.91−174.97 Ma) and the rise of Aleyrodomorpha (173.1 Ma, CI = 166.04−187.11 Ma). Notably, this geological period is estimated to correspond to the rise of Cicadidae (202.70 Ma, CI = 180.13−256.91 Ma). In both Sternorrhyncha and Heteroptera, several short-lifespan families are originating and declining (figure 2; electronic supplementary material, table S2).

The Cretaceous is the most significant period of major radiation of the extant Hemiptera (figure 2A–C). In Fulgoromorpha, Heteroptera and Sternorrhyncha, most of the extant families have their origin estimated during this period (electronic supplementary material, table S2). On the other hand, in Cicadomorpha and Coleorrhyncha, the Cretaceous appears to be a period of extinctions (e.g. Karabasiidae, Hoploridiidae and Progonocimidae; figure 2A,C). In Cicadomorpha, only one family originated during this geological period with the Cercopidae (68.61 Ma, CI = 66.01−73.75 Ma), whereas all Triassic to Jurassic ‘ancient’ families belonging to Clypeata went extinct (electronic supplementary material, table S2).

Lastly, during the Palaeogene, a small radiation occurred in Fulgoromorpha (figure 2), characterized by the origination of multiple planthopper families. In Heteroptera, numerous stink bug (Pentatomorpha) families originated during the Palaeocene–Eocene, whereas very few originations occurred for the Nepomorpha and Cimicomorpha families during this period (electronic supplementary material, table S2). In Sternorryncha, numerous extinctions are recorded during the Palaeogene, with multiple short-lived families (figure 2). No Cicadomorpha went extinct during the Palaeogene, but multiple originations of treehoppers (Aetalionidae, Membracidae) and froghoppers (Aphrophoridae, Clastopteridae) are found before the Oligocene–Miocene boundary (figure 2, electronic supplementary material, table S2).

We evaluated the impact of different ages (i.e. 325, 350, 386 and 407 Ma), used as the maximum boundary of the uniform prior on the root age, on our analyses of the origin and extinction ages for families (electronic supplementary material, figure S2). Our statistical tests do not detect a significant difference in our results (*p*-values > 0.05; electronic supplementary material, figure S2).

## 4. Discussion

The BBB model provides an alternative to traditional phylogenetic approaches that rely on fossil calibrations for divergence time estimates, a process that can significantly impact divergence time estimates [42]. In the latter, fossil assignment to a specific node is based on preserved apomorphic morphological characters [43]. However, confidently choosing fossils to calibrate a node (i.e. node-dating analyses) is often challenging, as fossil specimens may lack key diagnostic features, preserve only a limited set of traits, or fail to capture the full morphological variability of a group. An alternative approach, tip-dating analysis, where fossil taxa (tips) are associated with morphological data and temporal information and their placement and divergence time with other species are inferred using morphological clock models, requires comprehensive morphological datasets. This demand is one of the reasons such analyses remain relatively uncommon [44] (but see [23,28]). When a clade, such as Hemiptera, exhibited great diversity in deep past, especially at the family level, reconstructing its evolutionary history using a phylogenetic framework that excludes extinct taxa provides only a partial picture. This limitation affects both diversity dynamics estimates [6] and divergence dating analyses (figures 2 and 3). The BBB model helps address this issue by offering a more complete perspective on lineage origination and extinction timing. Crucially, it allows for the estimation of the origin and extinction ages of extinct groups, contributing to a more comprehensive understanding of Hemiptera’s evolutionary history.

### (a) The timing of origination and extinction of Hemiptera major lineages

The most comprehensive time-calibrated phylogenetic analyses of ‘Hemipterous’ insects suggest a Devonian origin of Hemiptera (386 Ma, CI = 354−427 Ma) [15]. Our estimates for the age of origin of Hemiptera are younger and converge towards an early Pennsylvanian age for the origin of the order, a result closer to previous estimates [14], and better aligning with the age of suborders (Sternorrhyncha, Cicadomorpha and Coleorrhyncha), and the oldest extinct Hemiptera families Archescytinidae, Aviorrhynchidae and Protoprosbolidae (figures 2 and 3; electronic supplementary material, tables S2 and S4). Interestingly, the estimated origin of Hemiptera is more precise than those of its suborders, reinforcing a previously observed trend: estimates become more precise, reflected in narrower 95% credibility intervals, when a greater number of species are included in the analysis [32–34]. This is the case here, as the analysis at the order level incorporates data from all hemipteran lineages. The comparatively older divergence estimates reported by Johnson *et al*. [15] may stem from imbalanced taxon sampling in their phylogenetic dataset—specifically, an overrepresentation of Hemiptera compared to Thysanoptera, Permopsocida and Psocodea. Such disparities can lead to biases in molecular clock analyses. Additionally, conflicting relationships at the root of ‘hemipterous’ insects may further complicate these estimates. In the literature, there is ongoing debate about whether Psocodea on one side, and (Permopsocida + (Hemiptera + Thysanoptera)) on the other side, form a monophyletic group Acercaria, a hypothesis we favour. Psocodea may instead be more closely related to Holometabola (e.g. [15,25,45,46]). In the case of an artifactual topology (i.e. Psocodea + Holometabola), divergence time estimates can become significantly skewed, often resulting in artificially older or younger age estimates. We anticipate that future studies, particularly those incorporating alternative data sources (i.e. non-transcriptomic data) and re-evaluating these relationships, will help clarify the phylogenetic relationships and divergence times near the root of the Hemiptera, and more generally, Acercaria.

The consensus of modern phylogenetic reconstructions places the Sternorrhyncha as the sister lineage to all other hemipteran groups [15,16]. Based on mitochondrial phylogenomics [16], Sternorrhyncha are estimated to have originated during the middle Permian (269 Ma, CI = 255−281 Ma; figure 3; electronic supplementary material, table S5), whereas transcriptomic phylogenomics [15] support an early Mississippian (356.37 Ma, CI = 322.42−396.66 Ma; figure 3; electronic supplementary material, table S5) origin of the clade. Our analysis based on the fossil record supports a late Carboniferous age (*ca* 302 Ma) for the origin of Sternorrhyncha offering an alternative intermediate scenario to molecular-based estimates.

The other major early diverging branch of the Hemiptera Tree of Life corresponds to Euhemiptera, encompassing the extant Cicadomorpha, Coleorrhyncha, Fulgoromorpha and Heteroptera, and the extinct Scytinopteromorpha and Ingruomorpha [5]. Considering only extant groups, the position of Coleorrhyncha (moss bugs) remains controversial. They were historically defined as the sister group of Heteroptera [45,47–51], but recent phylogenomics studies placed them as the sister group of Cicadomorpha + Fulgoromorpha [14,15,52]. Nevertheless, the position of Coleorrhyncha remains to be clarified as conflicting hypotheses are present in the literature [16,53]. The unresolved position of Coleorrhyncha within the Hemiptera Tree of Life, is likely to affect estimates of their time of origin. Our results support an old origin of this lineage, closer to the estimates of Johnson *et al*. [15], much older than estimates derived from molecular phylogenies based on few genes (263 Ma, CI = 254−281 Ma [54]; or mitogenomes (190 Ma, CI = 150−217 Ma [16]; figure 3; electronic supplementary material, tables S5 and S6).

Cicadomorpha have a fossil record dating back to the late Carboniferous, whereas the earliest known fossils of Fulgoromorpha are from the Permian [55]. Recent phylogenetic reconstructions support a sister-group relationship between these two clades, suggesting a gap in the fossil record of Fulgoromorpha between the clade’s inferred origin (i.e. the divergence from Cicadomorpha) and its first known fossil representatives. Our estimated origin of Fulgoromorpha during the Permian is also congruent with some molecular time divergence analyses [15,16,56]. This discrepancy may be attributed to the presence of ghost lineages but also to the fact that most Palaeozoic hemipteran fossils consist mostly of wings. Given the limitations of wing venation characters in confidently assigning fossils to specific clades, this may have hindered the accurate placement of certain specimens within Fulgoromorpha.

Heteroptera or true bugs are known since the Middle Triassic with Nepomorpha representatives [57]. Our analyses provided a Late Permian age for Heteroptera (254.45 Ma, CI = 248.02−281.09 Ma; electronic supplementary material, table S3), a younger age than phylogenetic estimates, which proposed a middle Permian to late Carboniferous age [14–16,58]. Nepomorpha (true aquatic bugs) are already well diversified during the Middle Triassic with the first occurrences of stem representatives of extant families such as Belostomatidae [59] and of the infraorder Cimicomorpha, with stem relatives to lace bugs or Tingoidea [60]. Therefore, their radiation probably occurred earlier in the Permian. Accurately estimating the divergence times of major heteropteran lineages remains a challenge due to a significant mismatch between the fossil record and molecular-based estimates. For example, phylogenomic analyses suggest that Enicocephalomorpha (unique-headed bugs) and Gerromorpha (semi-aquatic bugs) originated during the Permian [15,16,61], yet their earliest known fossils date only to the Cretaceous and Jurassic, respectively [62]. While newer approaches, such as the BBB model, help to better contextualize these discrepancies, closing these gaps will ultimately require the discovery of new Permian and Triassic fossil deposits or a re-examination of existing localities known to contain Hemiptera specimens.

### (b) Hemiptera originations and extinctions in deep time

Our analyses estimated a radiation of hemipteran families during the Permian, marking the emergence of multiple lineages within Cicadomorpha, Sternorrhyncha, and the now-extinct Scytinopteromorpha (figure 2B). During this period, 27 families originated, and 23 went extinct by the end of the Middle Triassic (electronic supplementary material, table S2; figure 2B,C). However, pinpointing the exact timing of the extinction of Palaeozoic Hemiptera fauna remains challenging due to the scarcity of fossil-bearing outcrops near the Permo-Triassic boundary [8,63]. While most phylogenetic-based studies suggest that major extant hemipteran lineages arose during the Permian [15,56,64], these studies do not account for now-extinct groups. Our BBB uncovered numerous radiations of extinct sets of taxa (e.g., Ingruomorpha, Progonocimicomorpha, Prosbolomorpha, Scytinopteromorpha), revealing a more complex evolutionary pattern and underscoring the importance of fossil-based approaches in reconstructing lineage histories (figure 2).

Furthermore, the highest peak in family-level diversification occurred during the Early Cretaceous (figure 2B), coinciding with the radiation of all modern hemipteran lineages (figure 2A). Notably, 19 of the 30 extant planthopper families, along with the majority of Sternorrhyncha and Heteroptera families, are estimated to have originated during this period (figure 2A; electronic supplementary material, table S3). These results align with phylogenetic studies indicating that many extant hemipteran groups emerged and diversified in the Cretaceous [56,65,66] as well as recent fossil-based analyses suggesting that Hemiptera, at the family level, diversified during mid-Cretaceous (electronic supplementary material, figure S3) [6,9]. Additionally, the mid-Cretaceous was also a major period of hemipteran extinctions, particularly among Palaeozoic/Triassic ‘relic’ lineages (e.g. Prosbolidae, Progonocimicidae) and specialized Cretaceous groups within Fulgoromorpha, Heteroptera and Sternorrhyncha (figure 2C).

Our results might indicate support for the hypothesis that these extinctions were driven by past environmental changes—particularly the shift in dominance in environments between angiosperms and gymnosperms—and the rise of other phytophagous insect lineages, which thrived alongside and co-evolved with angiosperm plants [6,67]. In particular, relic lineages that had evolved in association with non-angiosperm plants appear to have struggled to adapt to angiosperm-dominated ecosystems (figure 2A) [6]. Even when some groups managed to adapt, they may have been outcompeted by newly emerging hemipteran lineages or other phytophagous insects better suited to exploit these changing environments. While this pattern seems consistent across several insect clades [6,34,68,69], it remains essential to identify new fossiliferous deposits from the Maastrichtian, Campanian and Palaeocene to gain a clearer understanding of this pivotal phase in insect evolutionary history.

### (c) The relevance of the BBB model for dating diversified lineages in deep-time

The BBB model has a high potential for deciphering the evolutionary history of groups such as Coleorrhyncha (moss bugs), which are more diverse in deep time than today. Moss bugs, a relic clade, are today represented only by one extant family, the Peloridiidae, with 17 genera and 38 species [54]. However, Coleorrhyncha were probably more diverse in the past, possibly originating during the Permian, with four putative extinct families and 33 genera [40,70].

In phylogenetic analyses, the extant family Peloridiidae is typically the only lineage included to estimate divergence times for Coleorrhyncha, which introduces a clear bias. As a result, this group appears much younger than it likely is, with a total group estimated to originate in the Late Cretaceous [54]. This discrepancy stems from dating the crown age of Peloridiidae rather than the age of the Coleorrhyncha total group. Such limitations can significantly distort our understanding of the evolutionary history of Hemiptera. By incorporating extinct diversity, the BBB model allows for more accurate estimates of the age of the Coleorrhyncha total group.

An alternative to the BBB model for re-estimating the origin of this clade is a total-evidence dating approach [27,28], which integrates both morphological and molecular data from extant and extinct taxa to infer divergence times within Hemiptera. While this method holds great potential, its implementation is complex, requiring extensive morphological character matrices and substantial computational resources [28,30]. These practical considerations have limited its widespread application thus far. In contrast, the BBB model is less computationally demanding and more easily applicable across diverse lineages.

### (d) Limits of the study

Estimating the origin and extinction ages of a diverse lineage like Hemiptera is inherently challenging and requires a robust and well-resolved systematic framework. However, Hemiptera systematics, especially the relationships between extinct and extant lineages, remain poorly understood as most of the classification is based on fossil tegmina (preserving a limited number of diagnostic characters). A major challenge arises from the potential paraphyly of several fossil families, which may include stem-group members of other clades. For instance, some taxonomic frameworks place putative stem members of the Karabasiidae into the family Progonocimicidae, complicating accurate estimates of the origin of both groups. More broadly, the hypothesized relationships between extant Coleorrhyncha and extinct lineages such as Hoploridiidae, Karabasiidae and Progonocimicidae lack clear supporting apomorphies [40]. As a result, estimates of group origin and extinction ages using the BBB model, which relies on predefined taxonomic classifications, can be compromised by uncertainties in lineage delineation. Such uncertainties directly affect the vector representing species diversity over time, potentially leading to biassed estimates for taxonomic groups that are not monophyletic. Given the extensive taxonomic—including the discovery of new specimens and the study of microstructures, promising for the discovery of new diagnostic characters—and phylogenetic works required to resolve these taxonomic uncertainties, the most reasonable approach at present is to adopt a conservative strategy, relying on a relatively up-to-date and pre-established framework, encompassing fossil and extant lineages, while remaining aware of its limitations [5].

The BBB model is independent of any phylogenetic assumptions regarding relationships between lineages [32]. However, phylogenetic context can provide valuable information for estimating the timing of clade origins. When a lineage emerges, its sister lineage simultaneously arises as both diverge from a shared common ancestor. While this relationship does not specify the exact age of the crown group, it remains informative for estimating the age of the total group. In our analyses, we detected discrepancies in total-group ages for sister lineages, notably in the superfamily Cicadoidea (Tettigarctidae + Cicadidae) [71,72]. Our estimates place the age of the Tettigarctidae total-group in the Early Triassic, whereas that of the Cicadidae is estimated in the Jurassic (electronic supplementary material, table S2). This suggests that early representatives of stem Cicadidae might be missing from the fossil record or that they might be incorrectly assigned to another clade (e.g. Tettigarctidae) because of the absence of reliable features (in the venation) to discriminate between families.

While the BBB model is not explicitly affected by Lagerstätten, it remains susceptible to ‘directional bias’ introduced by the structure of the fossil record. In particular, when a Lagerstätte contains a concentration of the oldest known species of extant or extinct clades, or the youngest species of extinct clades, it can skew origin and extinction estimates toward the age of that deposit (figure 2A). For example, we observe a clustering of estimated ages for several Coccidomorpha lineages around the age of the Burmese amber (figure 2A). Although the BBB model has been shown to perform robustly under conditions of strong rate heterogeneity and typical preservation gaps in the insect fossil record [32], there are currently limited means to fully correct for this type of bias.

These limitations are not exclusive to the BBB model and can also impact traditional tree-based approaches and highlight the importance of careful interpretation when investigating events that happened in deep time. Whenever possible, we recommend comparing phylogenetic estimates with those derived from the BBB model or other fossil-based approaches, and vice versa, to construct a more comprehensive and reliable depiction of lineage evolutionary history.

## 5. Conclusion

We reconstructed a comprehensive evolutionary time scale for Hemiptera, a highly diverse lineage with a complex evolutionary history. Our results demonstrate that the fossil record is indispensable for understanding Hemiptera diversification and origin. We provide the first detailed estimates of the times of origin and extinction of all Hemiptera families with fossil representatives, as well as higher taxonomic ranks (i.e. superfamilies and suborders). Conducting such extensive dating at the scale of Hemiptera would have been extremely challenging, if not impossible, within a strictly phylogenetic framework, given the numerous extinct lineages classified within the order. We advocate for the broader application of the BBB model to large datasets like the Hemiptera fossil record, as it provides crucial insights into diversification and extinction patterns, especially regarding the timing of these events. Notably, our results highlight and confirm a mid-Cretaceous Hemiptera turnover, during which many new hemipteran families emerged while older, relic and specialized lineages underwent near-synchronous extinction. Ultimately, our study establishes a chronological framework for the origin and diversification of Hemiptera lineages, offering benchmarks that will be further refined with ongoing fossil discoveries and methodological advancements.

## Data Availability

The original dataset is extracted from Boderau et al. [6] and available in the Figshare digital repository [73]. The electronic supplementary material are available in the Figshare digital repository [74]. PyRate and rootBBB are freely available on Github (https://github.com/dsilvestro/PyRate; https://github.com/dsilvestro/rootBBB). The custom scripts of rootBBB working on multi-threading and GPU are available upon request to the first author. Supplementary material is available online [75].
